# Divergent Neuroinflammatory Regulation of Microglial TREM Expression and Involvement of NF-κB

**DOI:** 10.3389/fncel.2017.00056

**Published:** 2017-03-02

**Authors:** Rosie Owens, Kathleen Grabert, Claire L. Davies, Alessio Alfieri, Jack P. Antel, Luke M. Healy, Barry W. McColl

**Affiliations:** ^1^The Royal (Dick) School of Veterinary Studies, University of EdinburghMidlothian, UK; ^2^Neuroimmunology Unit, Montreal Neurological Institute, McGill UniversityMontreal, QC, Canada

**Keywords:** microglia, neuroinflammation, myeloid cells, NF-κB, TREM2, lipopolysaccharide (LPS), microglial activation

## Abstract

The triggering receptor expressed on myeloid cells (TREM) family of proteins are cell surface receptors with important roles in regulation of myeloid cell inflammatory activity. In the central nervous system, TREM2 is implicated in further roles in microglial homeostasis, neuroinflammation and neurodegeneration. Different TREM receptors appear to have contrasting roles in controlling myeloid immune activity therefore the relative and co-ordinated regulation of their expression is important to understand but is currently poorly understood. We sought to determine how microglial TREM expression is affected under neuroinflammatory conditions *in vitro* and *in vivo*. Our data show that microglial *Trem1* and *Trem2* gene expression are regulated in an opposing manner by lipopolysaccharide (LPS) *in vitro* in both adult murine and human microglia. LPS caused a significant induction of *Trem1* and a contrasting suppression of *Trem2* expression. We also observed similar divergent *Trem1* and *Trem2* responses *in vivo* in response to acute brain inflammation and acute cerebral ischaemia. Our data show that inhibition of NF-κB activation prevents the LPS-induced alterations in both *Trem1* and *Trem2* expression *in vitro* indicating NF-κB as a common signaling intermediate controlling these divergent responses. Distinct patterns of microglial *Trem1* induction and *Trem2* suppression to different Toll-like receptor (TLR) ligands were also evident, notably with *Trem1* induction restricted to those ligands activating TLRs signaling via TRIF. Our data show co-ordinated but divergent regulation of microglial TREM receptor expression with a central role for NF-κB. Neuroinflammatory conditions that alter the balance in TREM expression could therefore be an important influence on microglial inflammatory and homeostatic activity with implications for neuroinflammatory and neurodegenerative disease.

## Introduction

The triggering receptor expressed on myeloid cells (TREM) family of proteins comprises a group of cell surface innate immune receptors of the immunoglobulin superfamily that are expressed on various myeloid cell populations throughout the body, including microglia in the brain (Colonna, 2003; Colonna and Wang, 2016). The TREM gene cluster, located on human chromosome 6 and mouse chromosome 17, includes genes encoding TREM1 and TREM2 (human and mouse), and TREM3 (mouse only). The TREM-like genes, *Treml1* and *Treml2* which encode the proteins TREM-like transcript-1 (TLT-1) and TLT-2 also form part of this cluster. TREM proteins are structurally related, containing a single extracellular variable-type immunoglobulin domain and a transmembrane domain incorporating a charged lysine residue enabling association with the adaptor protein DAP12 that is obligatory for signal transduction (Klesney-Tait et al., 2006). Growing evidence supports a role for TREMs in the regulation of innate immune responses in various tissues, including the CNS (Sharif and Knapp, 2008). Moreover, despite sharing the requirement for signaling through DAP12 and its ITAM motif, it is becoming clear that TREM1 and TREM2 can have contrasting effects on immune regulation (Sharif and Knapp, 2008). Whereas TREM1, largely studied outside the CNS, has been shown to amplify inflammation in a number of models (Bouchon et al., 2000, 2001; Netea et al., 2006; Hommes et al., 2014, 2015), TREM2 appears to restrain myeloid cell responses to various inflammatory stimuli (Hamerman et al., 2006; Turnbull et al., 2006; Ito and Hamerman, 2012). Accordingly, the balance in signaling through these receptors is likely important for influencing the strength of innate immune activity.

The importance of TREM function in the CNS was first highlighted by the discovery of Nasu-Hakola disease, also known as Polycystic Lipomembranous Osteodysplasia with Sclerosing Leukoencephalopathy (PLOSL) (Hakola, 1972; Nasu et al., 1973). This is a fatal presenile dementia, also presenting with bone pathology, caused by homozygous loss of function mutations in TREM2 (or DAP12) affecting the microglial and ostecoclast myeloid lineages (Paloneva et al., 2000, 2001, 2002). Recent studies have also revealed that homozygous TREM2 mutations cause a frontotemporal dementia-like syndrome in the absence of bone pathology (Giraldo et al., 2013; Guerreiro et al., 2013a; Le Ber et al., 2014). Moreover, TREM2 mutations are associated with an increased risk of developing Alzheimer's disease (Guerreiro et al., 2013b; Jonsson et al., 2013), frontotemporal dementia (Thelen et al., 2014) and amyotrophic lateral sclerosis (Cady et al., 2014). Studies using animal models of neurodegeneration have also suggested disease-modifying effects of TREM2 (Piccio et al., 2007; Jiang et al., 2014; Jay et al., 2015; Wang et al., 2015). Mechanisms underlying TREM2-linked neurodegeneration are unclear but may involve impaired intracellular trafficking of TREM2 and deficiencies in microglial phagocytosis, reactivity and neuro-immune regulation (Colonna and Wang, 2016).

Regulation of TREM gene and protein expression will influence the extent and balance of signaling through these receptors and may be important for their effects on inflammation and disease. Elevated TREM2 gene or protein expression has been reported in animal models of chronic neurodegeneration (Jay et al., 2015; Wang et al., 2015) and in human post-mortem AD tissue (Lue et al., 2015), although the extent to which this simply reflects the marked proliferation of microglia that occurs in these conditions is unknown. In contrast, there is negligible data on TREM1 expression in the CNS. TREM1 is expressed on myeloid cells outside the brain including neutrophils, monocytes, macrophages and dendritic cells (Sharif and Knapp, 2008). Stimulation of macrophages with Toll-like receptor (TLR) ligands caused contrasting changes in TREM1 (increased) (Bouchon et al., 2001; Murakami et al., 2007; Zeng et al., 2007) and TREM2 (decreased) (Turnbull et al., 2006) expression. However, the coordinated regulation of TREM1 and TREM2 expression in microglia under neuroinflammatory conditions has not been studied previously. Furthermore, although signaling pathways triggered by TREM1 or TREM2 receptor binding have been described (Arts et al., 2013; Colonna and Wang, 2016), the intracellular mediators influencing transcriptional regulation of TREM genes are not well understood, particularly in microglia.

In the present study, we sought to determine the comparative regulation of TREM receptor expression in response to microglial activation and the signaling mechanisms involved. We show that microglial TREM1 and TREM2 expression is regulated in an opposing manner *in vitro* and *in vivo* and that NF-κB activation is a common intermediate governing this divergent regulation.

## Materials and methods

### Mining of microarray datasets for *Trem* analysis

To assess microglial *Trem* family expression at baseline in the adult mouse brain, we mined expression data for selected genes from microarray datasets we generated previously from purified microglia or mixed cell brain homogenates (Grabert et al., 2016). Datasets are publicly accessible at the NCBI gene Expression Omnibus (GEO) with accession code GSE62420 (http://www.ncbi.nlm.nih.gov/geo/GSE62420).

### Mice

Male 8–12 week old mice (C57BL/6J, Charles River Laboratories) were used in all experiments. Mice were maintained under specific pathogen-free conditions and a standard 12 h light/ dark cycle with unrestricted access to food and water. Mice were housed in individually ventilated cages in groups of up to five mice and were acclimatized for a minimum of 1 week prior to procedures. All procedures involving live animals were carried out under the authority of a UK Home Office Project License in accordance with the “Animals (Scientific Procedures) Act 1986” and Directive 2010/63/EU and were approved by The Roslin Institute's Animal Welfare and Ethics Committee.

### Adult mouse microglial isolation and culture

Microglia were isolated and cultured as described previously (Grabert et al., 2016). Briefly, mice were perfused transcardially with saline and the brain finely minced by scalpel blade in ice-cold Hanks Balanced Salt Solution HBSS (Sigma-Aldrich), centrifuged (400 g, 5 min, 4°C) resuspended and incubated for 1 h at 37°C in digestion cocktail (50 U/ml collagenase, 8.5 U/ml dispase, 100 ug/ml Nα-Tosyl-L-lysine chloromethyl ketone hydrochloride, 5 U/ml DNaseI in HBSS). Tissue was dissociated using a Dounce homogeniser and the enzymatic reaction terminated by addition of equal volume HBSS containing 10% fetal bovine serum. Homogenates were centrifuged (400 g, 5 min, 4°C) and pellets resuspended in 35% Percoll (GE Healthcare, Sweden), overlaid with HBSS then centrifuged (800 g, 45 min, 4°C). The cell pellet enriched with microglia was resuspended in separation buffer (0.5% bovine serum albumin, 2 mM EDTA in PBS). The cell suspension was incubated with anti-CD11b microbeads (Miltenyi Biotec, UK) for 15 min at 4°C then applied to a magnetic LS column (Miltenyi Biotec) and cells retained on the column (microglia) were flushed. The microglial suspension was centrifuged (400 g, 5 min, 4°C) and resuspended in DMEM/F12 (Invitrogen) containing 10% FBS and 1% penicillin/streptomycin. Cells were seeded in wells pre-coated with poly-L-lysine at a density of 2 × 10^5^ cells/ml and cultured at 37°C in a humidified incubator under 5% CO_2_. Media was replaced after 48 h in culture and cell stimulations performed after 72 h in culture by which time cells developed a more ramified morphology characteristic of microglia. Cells were stimulated for 24 h with LPS (1 μg/ml), IL-4 (20 ng/ml) or DPBS.

### BV2 microglia culture

Although there are limitations of using any cell line, the utility of immortalized BV2 microglia as a suitable substitute for primary microglia has been shown (Henn et al., 2009). BV2 cells were cultured in DMEM (Invitrogen) supplemented with 5% fetal bovine serum and 1% penicillin/streptomycin at 37°C in a humidified incubator under 5% CO_2_. Cells were seeded at a density of 2.5 × 10^5^ cells/ml 24 h before stimulations at which point cells were >90% confluent. Prior to stimulation, cells were washed twice with DPBS (Invitrogen) and cultured in serum-free DMEM for at least 1 h. Cells were stimulated for 24 h with one of the following: LPS (*E. coli* 0127:B8 1 μg/ml; Sigma-Aldrich); recombinant mouse IL-4 (20 ng/ml; R&D Systems); Pam3CSK4 (5 μg/ml; Invivogen); polyinosinic-polycytidylic acid (poly(I:C)) (50 μg/ml; Sigma-Aldrich); CpG oligodeoxynucleotides (CpG ODN) (1 μM; Enzo Life Sciences); or DPBS. In certain experiments, cells were pre-incubated with inhibitors (Merck Millipore) of IκB kinase (BMS-345541, 1 or 10 μM), PI3 kinase (LY294002, 10 μM), ERK1/2 kinase (MEK1/2) (PD98059, 10 μM), p38 MAP kinase (SB203580, 10 μM), c-Jun N-terminal kinase (SP600125, 20 μM) or equivalent volume of DMSO for 30 min prior to LPS stimulation.

### Human microglial isolation and culture

Human microglia were isolated from adult brain tissue using previously described protocols (Durafourt et al., 2013) under ethical approval by the McGill University Health Center Research Ethics Board (protocol ANTJ2001/1). Briefly, normal appearing cortical tissue was resected from pharmacologically intractable non-malignant cases of temporal lobe epilepsy. Tissue was cleaned extensively and mechanically dissociated. A single cell suspension was generated following gentle enzymatic digestion using trypsin and DNAse prior to passing through a nylon mesh filter. Single cell suspension underwent a Ficoll ultracentrifugation step to remove myelin. Dissociated cells were centrifuged, counted, and plated at 2 × 10^6^ cells/mL in MEM supplemented with 5% FBS, 0.1% penicillin/streptomycin and 0.1% glutamine. Microglia were grown for 3 days, collected and plated at 1 × 10^5^ cells/mL and maintained in culture for 6 d during which time cells were left untreated or stimulated. Cells were treated with IFN-γ (20 ng/ml) for 1 h followed by a 48 h treatment with LPS (*E coli* 0127:B8, 100 ng/ml) or with IL-4 (20 ng/ml) and IL-13 (20 ng/ml) together for 48 h.

### Human microglial gene expression analysis

Microglia gene expression profiles was measured using a Human Gene ST 2.0 Microarray Chip (Affymetrix). Raw intensity data files were normalized using the Robust Multichip Average (RMA) algorithm, averaged with Tukey's bi-weight average algorithm, and transformed to the log2 scale using the Affymetrix Expression Console. Data was exported from Expression Console into Affymetrix Transcriptome Analysis Console (TAC) software. GraphPad Prism 5 was used to perform statistical analyses (ANOVA and Bonferroni's multiple comparison Test) and plot individual gene expression. Data are expressed as Tukey's bi-weight average ± SD. The dataset is publicly accessible in NCBI's GEO with accession number GSE76737 (https://www.ncbi.nlm.nih.gov/geo/GSE76737).

### Intracerebral inflammation model

Mice were positioned in a stereotaxic frame (Stoelting, USA) under isoflurane anesthesia (with 0.2 L/min O_2_ and 0.5 L/min N_2_O). The skull was exposed via a midline incision and a small craniotomy was made overlying the left hemisphere 2 mm lateral to Bregma using an Ideal micro drill (tip diameter 0.8 mm) (Stoelting). Stereotaxic injections were performed using pre-calibrated glass microcapillary pipettes (Drummond Scientific, USA), previously pulled using a vertical electrode puller (Model PP830, Japan). Co-ordinates for intrastriatal injection were 2 mm lateral to Bregma (left hemisphere) and 2.5 mm below the brain surface (Franklin and Paxinos, 2007). One microliter LPS (*E. coli* 0127:B8, 5 mg/ml) or PBS was injected at a rate of 0.5 μL/min. The wound was sutured and topical local anaesthetic (EMLA lidocaine/prilocaine) applied. Mice were recovered for 24 h.

### Middle cerebral artery occlusion experimental stroke model

Middle cerebral artery occlusion (MCAO) was performed under isoflurane anesthesia (with 0.2 L/min O_2_ and 0.5 L/min N_2_O) by insertion of a 6-0 nylon monofilament with a 2 mm coated tip (210 μm diameter; Doccol, USA) through the external carotid artery and advanced through the internal carotid artery to occlude the middle cerebral artery (MCA). The filament was withdrawn after 40 min to allow reperfusion, the neck wound sutured and the animals recovered. Topical local anaesthetic (EMLA lidocaine/prilocaine) was applied to the wound. For sham surgery, the filament was advanced to the MCA and immediately retracted. Sham-operated animals remained anaesthetized for 40 min and recovered as above. Core body temperature was maintained at 37 ± 0.5°C throughout the procedure with a feedback controlled heating blanket (Harvard Apparatus, UK). Mice were recovered for 24 h.

### Brain cell suspension preparation

24 h after intracerebral LPS injection or MCAO, mice were perfused transcardially under isoflurane anesthesia as above with DEPC-treated (0.1%) saline to remove blood and the hemisphere ipsilateral to intracerebral injection or MCAO was dissected. Brain cell suspensions were prepared as above for microglial isolation except after Percoll centrifugation cell pellets were resuspended in FACS buffer for immediate flow cytometry staining or in RNAprotect Cell Reagent (Qiagen) for storage at −80°C.

### RNA extraction and cDNA synthesis

For RNA extractions from BV2 cells and isolated adult mouse microglia, media was removed and cells incubated with Trizol for 2 min at room temperature and mixed by pipetting then incubated for a further 5 min. For brain cell suspensions prepared after intracerebral inflammation and stored in RNAprotect Cell Reagent (Qiagen), cell suspensions were thawed and centrifuged (5,000 g, 5 min, 4°C). The supernatant was discarded and the cell pellet incubated with Trizol as above. Lysates were incubated with 1-bromo-3-chloropropane (BCP) for 2 min and centrifuged (12,000 g, 5 min, 4°C) then the upper aqueous phase was removed and the previous step repeated. The aqueous phase was then incubated with 1 μl co-precipitant linear acrylamide and 250 μl isopropanol before centrifugation (12,000 g, 10 min, 4°C). The supernatant was removed and the RNA pellet washed in 75% ethanol. The RNA suspension was then centrifuged (7,500 g, 5 min, 4°C), air-dried and resuspended in DNase/RNase-free water. For brain homogenates prepared after MCAO, RNA was extracted using the RNEasy Mini Kit (Qiagen) according to manufacturer's instructions. DNase treatment of all RNA samples was performed using the DNA-free kit (Ambion) according to manufacturer's instructions. Concentration of RNA samples was determined by Nanodrop 1000 (Thermo Fisher Scientific) measurement. cDNA was synthesized from DNase-treated RNA samples using Superscript III Reverse Transcriptase (Life Technologies) according to manufacturer's instructions.

### Quantitative PCR

Primers were designed using Primer BLAST (http://www.ncbi.nlm.nih.gov/tools/primer-blast/) and validated to ensure adequate efficiencies. Primer sequences were as follows: *Arg1* forward 5′-GGAGACCACAGTCTGGCAGTTGGA-3′; *Arg1* reverse 5′-GGACACAGGTTGCCCATGCAGA-3′; *Gapdh* forward 5′-TGCATCCACTGGTGCTGCCAA-3′; *Gapdh* reverse 5′-ACTTGGCAGGTTTCTCCAGGCG-3′; *Nos2* forward 5′-GGAGACCACAGTCTGGCAGTTGGA-3′; *Nos2* reverse 5′-AGGTCGATGCACAACTGGGTGAAC-3′; *Trem1* forward CTGGTGGTGACCAAGGGTTC; *Trem1* reverse CTTGGGTAGGGATCGGGTTG; *Trem2* forward 5′-CTGCTGATCACAGCCCTGTCCCAA-3′; *Trem2* reverse 5′-CCCCCAGTGCTTCAAGGCGTCATA-3′; *Gapdh* was used as the housekeeping gene. cDNA was mixed with forward and reverse primers, ROX reference dye and Platinum SYBR Green qPCR Supermix-UDG in DNase/RNase-free water and qPCR cycles performed on a Stratagene Mx3005P thermocycler (Agilent) as follows: hot-start denaturation cycle 95°C for 10 min, 40 cycles of amplification at 95°C for 15 s, 60°C for 20 s and 72°C for 1 min, followed by one cycle of 95°C for 1 min, 55°C for 30 s and 95°C for 30 s. Cycle threshold (Ct) values of target genes were normalized to *Gapdh* and data are expressed as fold change relative to control group using the 2^∧^ΔΔCt method.

### Flow cytometry

Media was removed from cultured BV2 cells and cells washed with DPBS then detached by incubation with trypsin for 30 s at 37°C. DMEM was added to neutralize trypsin and samples were centrifuged (400 g, 5 min) then pellets resuspended in FACS buffer (DPBS containing 0.1% low endotoxin BSA). Cell suspensions were added to a 96-well plate and low affinity Fc receptors blocked by incubation of cells with anti-CD16/32 antibody for 30 min. Plates were centrifuged (400 g, 5 min), supernatants discarded and cell pellets disrupted by gentle agitation of plates. Cells were incubated with the following fluorochrome-conjugated rat monoclonal antibodies for 30 min: anti-mouse F4/80-PerCP (clone BM8, 1 μg/ml, BioLegend); anti-mouse/human CD11b-APC-Cy7 (clone M1/70, 1 μg/ml, BioLegend); anti-mouse TREM2-FITC (clone 78.18, 1 μg/ml, Abd Serotec); anti-mouse TREM1-Alexa Fluor 647 (clone L5-B8.2A12.3A12, 5 μg/ml, Abd Serotec). Plates were centrifuged (400 g, 5 min), supernatants discarded and cells resuspended in FACS buffer. For flow cytometric analysis after MCAO, brain cell suspensions were added to a 96-well plate and stained with anti-mouse CD45-PE-Cy (clone 30-F11, 1 μg/ml, BioLegend), anti-mouse Ly6C-PerCP-Cy5.5 (clone HK1.4, 1 μg/ml, BioLegend) and anti-mouse TREM1-Alexa Fluor 647 (clone L5-B8.2A12.3A12, 5 μg/ml, Abd Serotec) following the above procedure. Data were acquired using a LSR Fortessa (BD Biosciences) and analyzed using Summit (Dako) or FlowJo software. Compensation was performed using single-labeled samples as references and positive regions of staining were defined based on unstained and/or isotype-stained controls.

### Experimental design and statistical analysis

Data from BV2 and adult mouse microglial are representative of at least three independent experiments each containing a minimum of triplicate technical replicates. Data from MCAO (*n* = 6 mice per group) and intracerebral inflammation models (*n* = 6 mice per group) are from a single experiment containing independent biological replicates (i.e., mice). One sample *t*-test was used to assess effects of microglial stimulation, MCAO or intracerebral inflammation on *Trem1* and *Trem2* expression by qPCR using the reference value of 1 for the PBS or sham control groups. One-way ANOVA with Bonferroni correction was used to assess effects of microglial stimulation on TREM2 protein expression by flow cytometry. Effects of IKK inhibition on *Trem1* and *Trem2* expression were assessed using one-way ANOVA with Dunnett's correction (LPS + DMSO as reference group). Effects of MCAO on microglial TREM1 expression after MCAO measured by flow cytometry were assessed using unpaired Student's *t-*test. Analysis was performed blinded to experimental treatment. Data were analyzed using GraphPad Prism and show mean ± SEM unless stated otherwise. *P* ≤ 0.05 was considered statistically significant.

## Results

### Steady-state microglial *Trem* family expression

**Figure 1:**
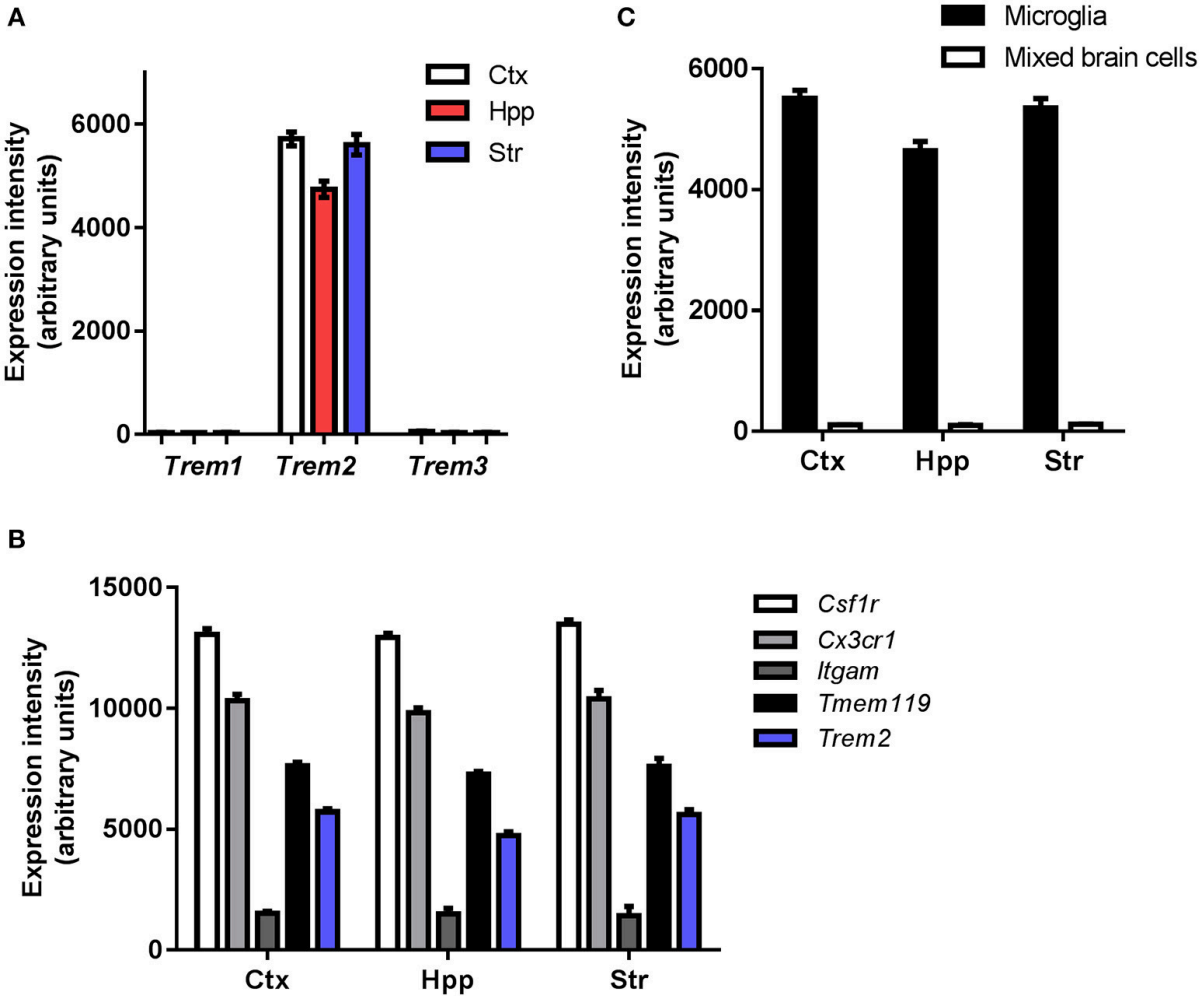
**Steady-state expression of *Trem* family genes in microglia. (A)** Microarray expression intensities of *Trem* family genes in freshly isolated adult murine microglia extracted from different forebrain regions. **(B)** Microarray expression intensities of *Trem2* in isolated microglia and mixed cell brain homogenates from different forebrain regions. **(C)** Microarray expression intensities of *Trem2* relative to other established genes highly expressed in microglia. Microarray data were re-analyzed from publicly available datasets (GSE62420) we generated previously (Grabert et al., 2016). Ctx, cerebral cortex; Hpp, hippocampus; Str, striatum.

To gain an initial understanding of steady-state microglial expression of *Trem* family members we first examined the pattern of gene expression in purified adult mouse microglia. *Trem2* expression was abundant in microglia from all forebrain regions examined and at markedly greater levels than *Trem1* and *Trem3*, both of which were expressed at negligible levels (Figure 1A). Comparison to established genes highly expressed in microglia (e.g., *Csf1r, Cx3cr1, Itgam, Tmem119*) gave an indication of the extent of microglial *Trem2* expression levels (Figure 1B). To assess if microglia are the predominant cellular source of *Trem2* expression in the brain, we compared expression in purified microglia and mixed brain cell lysates. Expression was substantially enriched in purified microglia and negligible expression was observed in mixed cell lysates (Figure 1C) thus showing that microglia are the predominant source of *Trem2* expression in the steady-state young adult brain. There is no functional ortholog of *Trem3* in humans where it is a pseudogene (TREM3 has similar actions to TREM1 in mice; Chung et al., 2002; Colonna, 2003) therefore we limited analysis in the remainder of the study to *Trem1* and *Trem2*.

### Polarization of mouse and human microglia differentially regulates *Trem* expression

**Figure 2:**
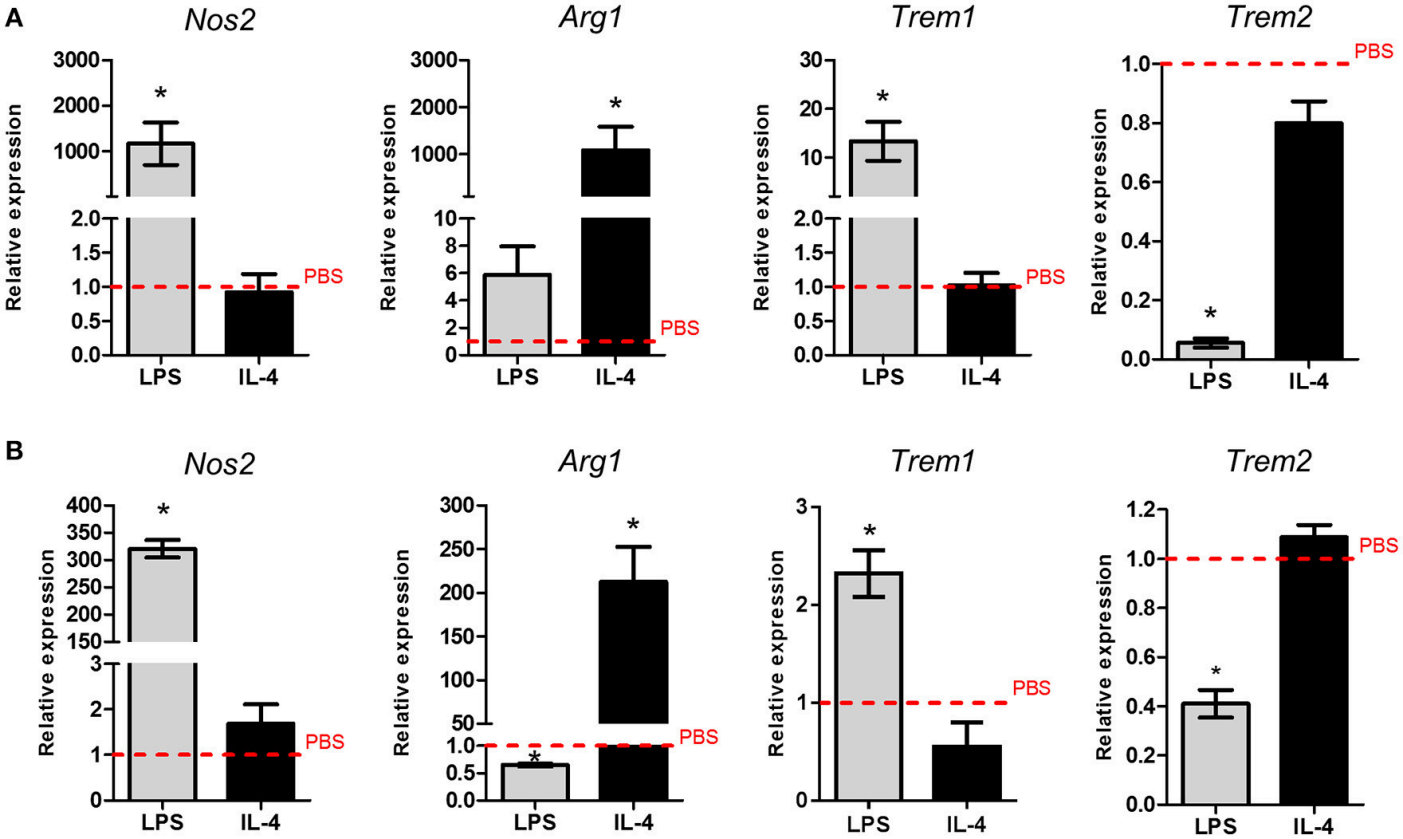
**Effects of polarized activation of murine microglia on *Trem1* and *Trem2* expression. (A)** Microglia were extracted from the adult mouse brain then cultured and stimulated for 24 h with lipopolysaccharide (LPS) or (IL-4) and gene expression measured by qPCR. **(B)** BV2 microglia were cultured and stimulated for 24 h with LPS or IL-4 and gene expression measured by qPCR. Data show mean ± SEM and are representative of three independent cultures each performed in triplicate. Data are expressed as fold-change in expression vs. PBS treatment (dashed red line). ^*^P < 0.05, one-sample *t*-test. LPS, lipopolysaccharide; IL-4, interleukin-4.

Next we examined the effect of *in vitro* activation of purified adult microglia on *Trem* expression. We chose lipopolysaccharide (LPS) and interleukin (IL)-4 as stimuli to induce contrasting states of activation. Marked induction of *Nos2* expression in response to LPS and *Arg1* expression in response to IL-4 validated that purified adult microglia could be polarized in the expected manner (Figure 2A). *Trem1* expression was significantly increased (~10-fold) in response to LPS but was unaffected by IL-4 stimulation (Figure 2A). In contrast, *Trem2* expression was suppressed by ~90% in response to LPS stimulation. Similarly to *Trem1* expression, *Trem2* was unaffected by IL-4 stimulation (Figure 2A). A similar pattern of responses of *Trem1* and *Trem2* to LPS and IL-4 stimulation were observed in the murine BV2 microglial cell line (Figure 2B), although we note the generally more responsive nature of the isolated adult microglia. These data indicate opposing regulation of *Trem1* and *Trem2* genes by LPS-induced microglial activation and more generally that the conditions under which microglia become activated differentially affect *Trem* responses.

**Figure 3:**
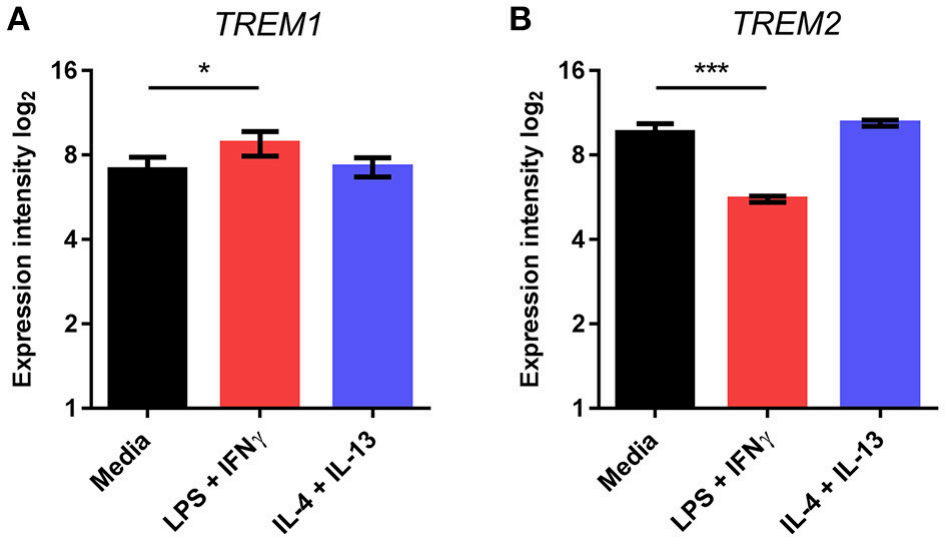
**Effects of polarized activation of human microglia on *Trem1* and *Trem2* expression**. Microglia were extracted from resected human brain samples then cultured and stimulated for 48 h with lipopolysaccharide (LPS) and interferon-γ in combination or interleukin-4 (IL-4) and IL-13 in combination. Expression of **(A)** *TREM1* and **(B)** *TREM2* were measured by microarray. Data are displayed as log_2_ values and show mean ± SD. ^*^P < 0.05, ^***^P < 0.001 one-way ANOVA with Dunnett's correction. LPS, lipopolysaccharide; IFNγ, interferon-γ; IL-13, interleukin-13; IL-4, interleukin-4.

We next tested if the pattern of *TREM* regulation was also observed in freshly isolated adult human microglia. Human microglia were activated using comparable stimulation protocols to mouse microglia that were previously validated (Durafourt et al., 2013). As in mouse microglia, we found that *TREM1* expression was significantly increased and *TREM2* expression significantly decreased in response to combined stimulation with LPS and interferon-γ (IFNγ) whereas there were no changes in response to combined IL-4 and IL-13 stimulation (Figures 3A,B).

### Differential effects of microglial polarization on TREM2 protein expression

**Figure 4:**
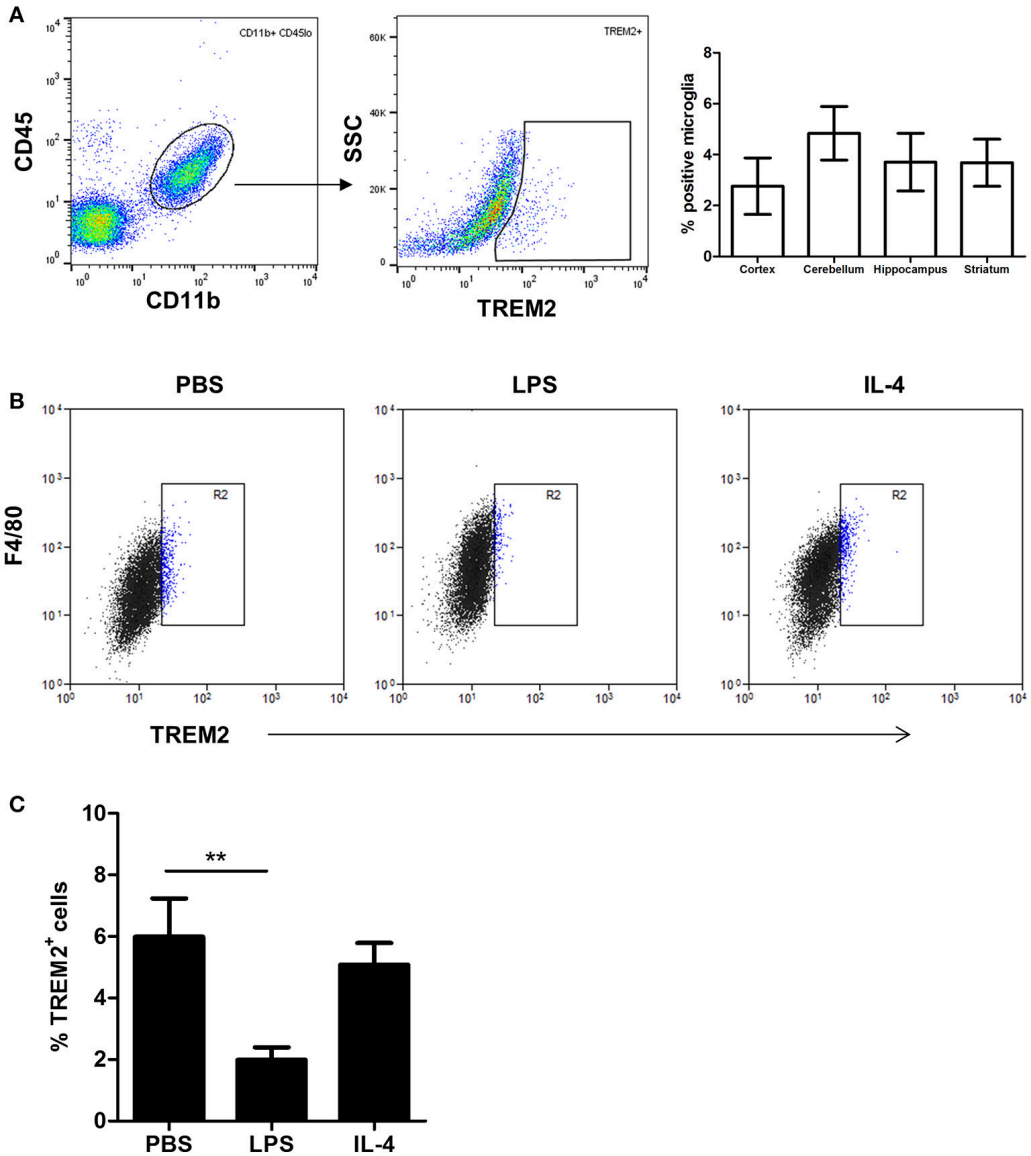
**Effects of polarized activation of murine microglia on TREM2 protein expression. (A)** Microglia were identified as CD11b^+^CD45^lo^ cells from murine brain cell suspensions prepared from different brain regions and surface TREM2 expression measured by flow cytometry. Data show proportion of microglia positive for TREM2 immunolabeling. **(B)** BV2 cells were stimulated with LPS or IL-4 for 24 h and cell surface TREM2 and F4/80 expression measured by flow cytometry. Plots are representative of four independent cultures. **(C)** Quantification of cell surface TREM2 expression in BV2 cells stimulated with LPS or IL-4 for 24 h by flow cytometry. Data show mean ± SEM and are representative of four independent cultures each performed in triplicate. ^**^P < 0.01 one-way ANOVA with Dunnett's correction. LPS, lipopolysaccharide; IL-4, interleukin-4.

Given the marked suppression of *Trem2* gene expression by LPS stimulation, we assessed if this effect extended to altered protein expression. Previous studies have shown that TREM2 protein is predominantly localized within intracellular vesicles of non-activated microglial cell lines (Sessa et al., 2004; Prada et al., 2006). Consistent with these data, using flow cytometry staining of non-permeabilised cells we found that only a small proportion (3–6%) of freshly isolated adult mouse microglia (Figure 4A) or cultured BV2 cells (Figure 4B) stained positively for TREM2. Stimulation with LPS or IL-4 increased the intensity of surface F4/80 expression (Figure 4B) confirming cells were activated as expected. LPS stimulation caused a significant reduction in the proportion of surface TREM2^+^ microglia whereas expression was unaffected in response to IL-4 (Figure 4C). Thus, the differential effects of microglial polarization on *Trem2* gene expression are also evident at the protein level.

### NF-κB is a common mediator of divergent microglial *Trem1* and *Trem2* expression

**Figure 5:**
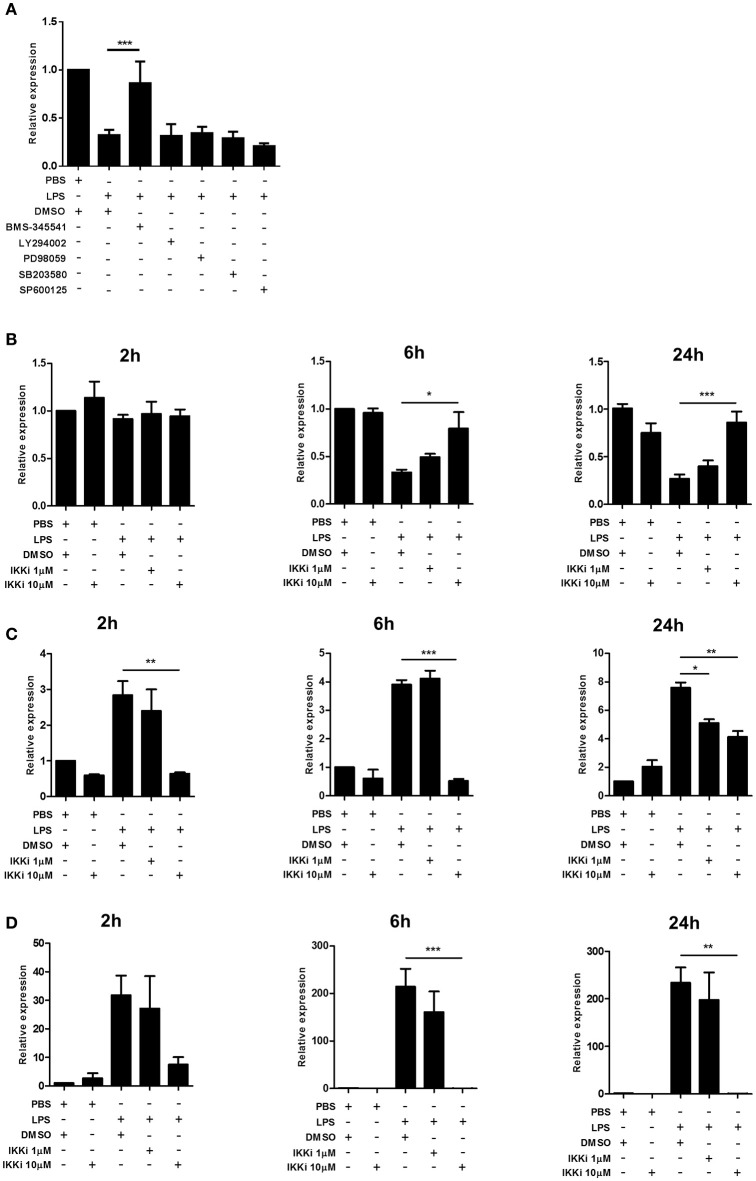
**Effects of NF-κB inhibition on *Trem1* and *Trem2* expression in activated murine microglia. (A)** BV2 cells were stimulated with LPS for 24 h with or without co-incubation with inhibitors of IκB kinase (BMS-345541), PI3 kinase (LY294002), ERK1/2 kinase (MEK1/2) (PD98059), p38 MAP kinase (SB203580), or c-Jun N-terminal kinase (SP600125) and expression of Trem2 measured by qPCR. (B-D) BV2 cells were stimulated for indicated duration with LPS with or without co-incubation with the IkB kinase inhibitor (IKKi) BMS-345541 and **(B)** *Trem2*, **(C)** *Trem1*, and **(D)** *Nos2* measured by qPCR. Data show mean ± SEM and are representative of two **(A)** or four **(B–D)** independent cultures each performed in triplicate. Data are expressed as fold-change in expression vs. PBS treatment. ^*^P < 0.05, ^**^P < 0.01, ^***^P < 0.001 one-way ANOVA with Dunnett's correction. LPS, lipopolysaccharide; IKKi, IκB kinase inhibitor.

We next explored the intracellular signaling mechanisms mediating the effect of LPS-induced microglial activation on *Trem2* expression. In view of the comparable responses of BV2 cells to freshly isolated adult human and mouse microglia observed above and the difficulties in acquiring large yields of fresh cells we limited the following experiments to BV2 cells. We focused on key components of the TLR signaling cascade activated in response to LPS stimulation. Initial screening using selective inhibitors of p38 MAPK, ERK1/2 kinase (MEK1/2), JNK, and PI3 kinase showed no impact on LPS-induced *Trem2* alterations (Figure 5A). In contrast, inhibition of the NF-κB pathway using the highly selective IKK inhibitor BMS-345541 (Burke et al., 2003) markedly attenuated the LPS-stimulated suppression of *Trem2* (Figure 5A). We found that LPS stimulation suppressed *Trem2* expression at 24 h and at 6 h but not 2 h after stimulation (Figure 5B). There was a concentration-dependent attenuation of the suppressive effects of LPS stimulation on *Trem2* expression at 6 h and 24 h with the higher concentration (10 μM) of BMS-345541 restoring expression to near baseline levels. We also determined if there was an important contribution of the NF-κB pathway to microglial *Trem1* regulation. IKK inhibition blocked the LPS-induced increase in *Trem1* expression 2 h after stimulation in a concentration-dependent manner and this effect was sustained to 6 and 24 h after LPS stimulation (Figure 5C). At 2 and 6 h, the high concentration (10 μM) of BMS-345541 almost completely inhibited the induction of *Trem1*. We also observed a potent concentration-dependent effect of IKK inhibition on blocking LPS-induced *Nos2* expression (Figure 5D). These data show that the NF-kB pathway is a common regulatory mechanism underlying the divergent effects of microglial activation on *Trem1* and *Trem2* expression.

### Microglial *Trem1* and *Trem2* regulation have contrasting TLR sensitivity profiles

**Figure 6:**
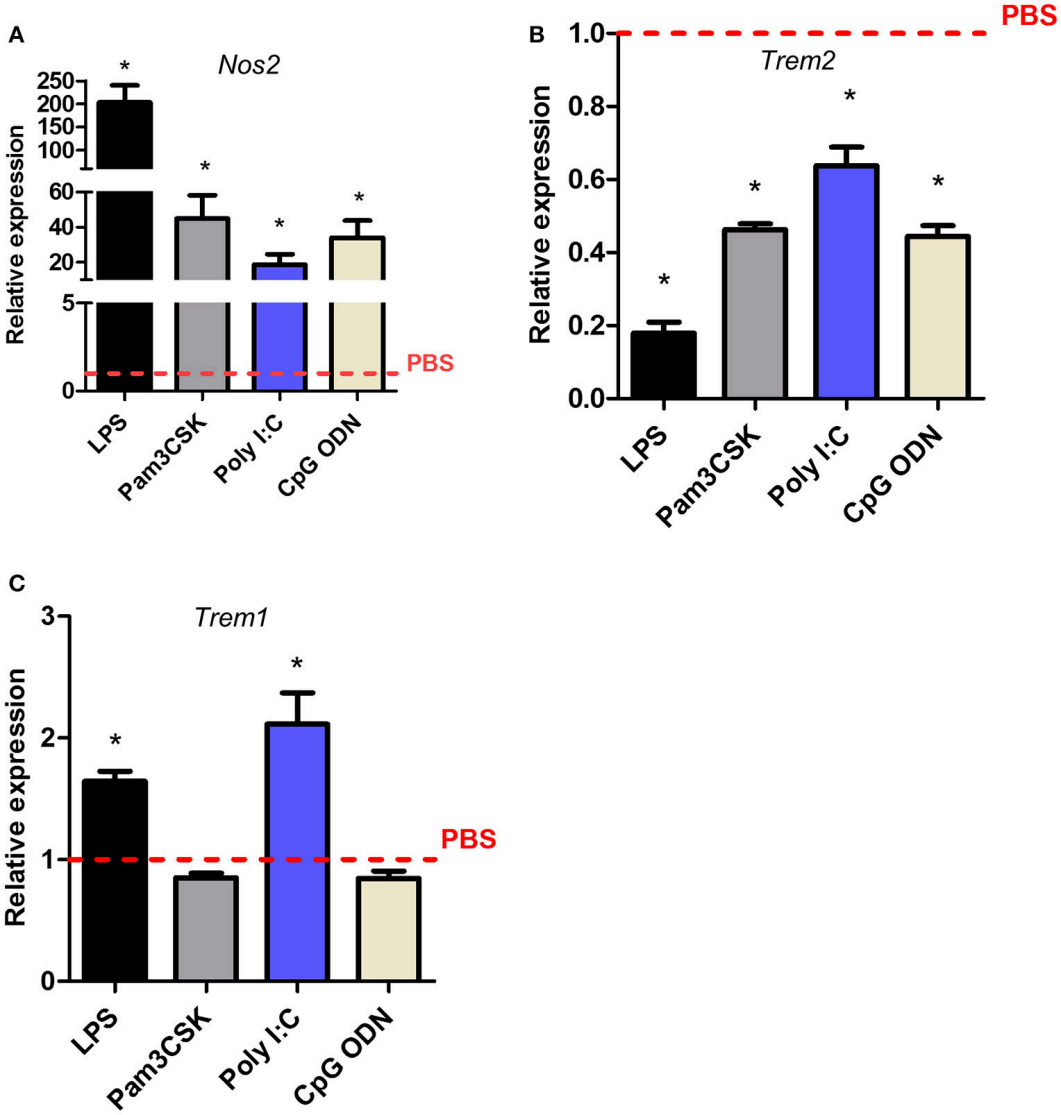
**Effects of Toll-like receptor subtype stimulation on *Trem1* and *Trem2* expression in murine microglia**. BV2 cells were stimulated with indicated ligands for 24 h and expression of **(A)** *Nos2*, **(B)** *Trem2*, and **(C)** *Trem1* measured by qPCR. Data show mean ± SEM and are representative of four independent cultures each performed in triplicate. Data are expressed as fold-change in expression vs. PBS treatment (dashed red line). ^*^P < 0.05, one-sample *t*-test. LPS, lipopolysaccharide; poly I:C, polyinosinic-polycytidylic acid, CpG ODN, CpG oligodeoxynucleotide.

LPS triggers intracellular signaling in microglia through binding to the TLR4 receptor. We next determined if *Trem* expression was affected in a similar manner when other TLRs were engaged. Pam3CSK, poly (I:C), LPS, and CpG oligodeoxynucleotide (CpG ODN) were used to stimulate TLR2/1, TLR3, TLR4, or TLR9 respectively. Induction of *Nos2* by all ligands confirmed biological activity and responsiveness of microglia to each stimulus (Figure 6A). *Trem2* expression was significantly suppressed by all TLR ligands although the magnitude varied (Figure 6B). Expression of *Trem2* was reduced to the greatest extent by LPS (~80%) and weakest suppression occurred in response to poly (I:C) (~35%) with intermediate effects induced by Pam3CSK and CpG ODN. In contrast to the largely consistent suppression of *Trem2* expression by activation of different TLRs, *Trem1* expression was affected in a TLR-specific manner. LPS, as observed above, and poly (I:C) both induced a significant increase in *Trem1* expression whereas there was no effect of Pam3CSK or CpG ODN (Figure 6C). These data show differences in the regulation of *Trem1* and *Trem2* dependent on the specific TLR engaged and suggestive of differences in the involvement of signaling intermediates upstream of NF-κB (see Discussion).

### Acute brain inflammation induces divergent changes in *Trem* expression *in vivo*

**Figure 7:**
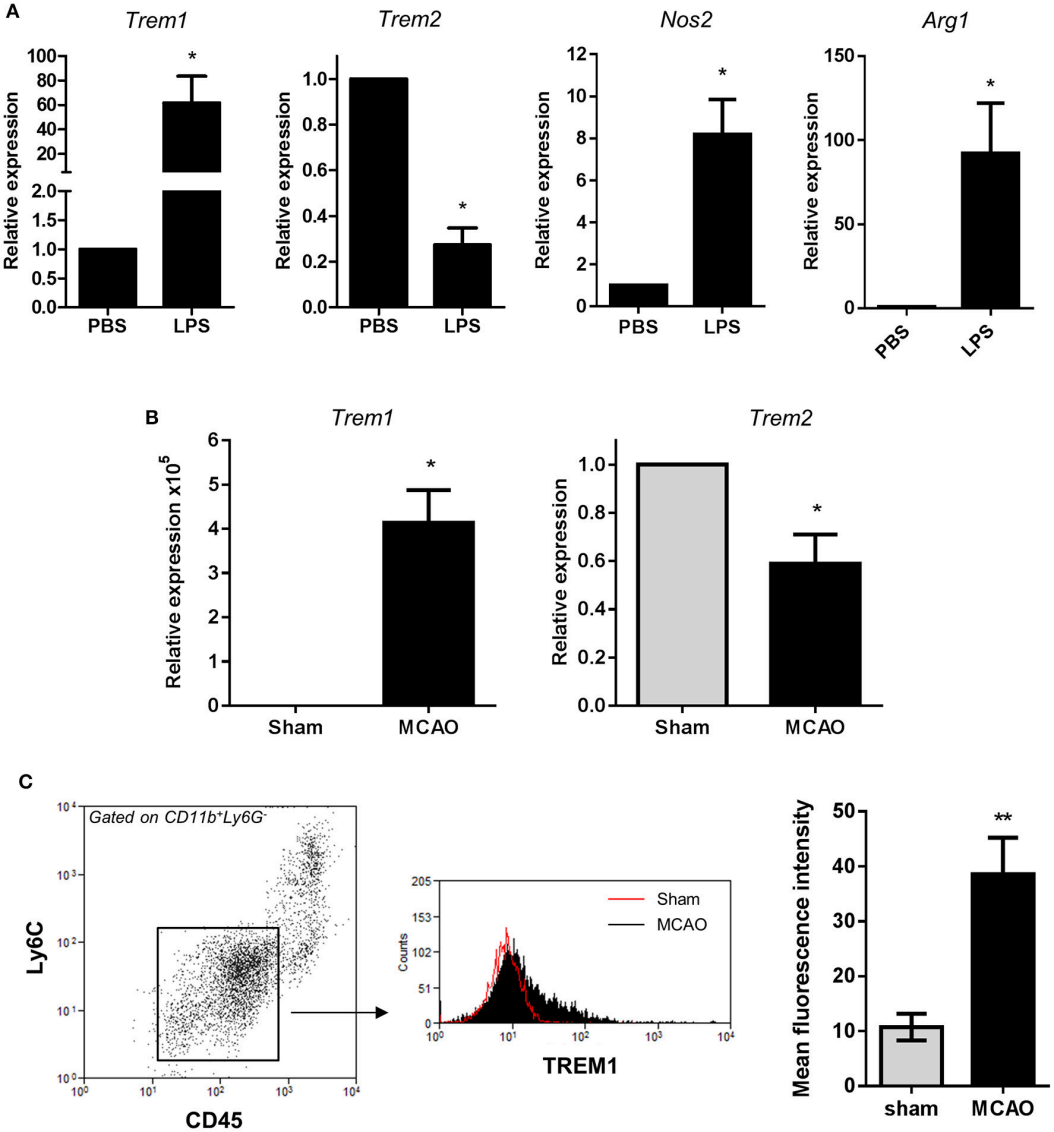
***Trem1*** **and *Trem2* expression *in vivo* in response to acute cerebral inflammatory challenges. (A)** Mice were challenged with LPS by intracerebral injection and expression of indicated genes measured in brain cell suspensions 24 h after challenge by qPCR. Data are expressed as fold-change in expression vs. PBS treatment. Data show mean ± SEM. ^*^P < 0.05, one-sample *t*-test, *n* = 6 per group **(B)** Transient focal cerebral ischaemia was induced in mice by middle cerebral artery occlusion (MCAO) and *Trem1* and *Trem2* expression measured in brain homogenates ipsilateral to occlusion 24 h after MCAO. Data are expressed as fold-change in expression vs. PBS treatment. Data show mean ± SEM. ^*^P < 0.05, one-sample *t*-test, *n* = 6 per group **(C)** Brain cell suspensions were prepared 24 h after MCAO and microglia identified by CD11b^+^CD45^lo^Ly6G^−^Ly6C^−^ expression. TREM1 cell surface expression was measured on microglia by flow cytometry. Flow cytometry plots are representative of six independent samples. Data show mean ± SEM. ^**^P < 0.01, Student's *t*-test, *n* = 6 per group.

To test if the divergent regulation of *Trem* expression described above *in vitro* was also evident *in vivo*, we used two models of acute brain inflammation. First, the expression of *Trem1* and *Trem2* was determined in brain cell extracts 24 h after intracerebral LPS injection in mice, a model that induces a localized intense inflammatory reaction. LPS induced a significant increase in *Trem1* expression and a contrasting suppression of *Trem2* expression (Figure 7A) thus matching the profile observed *in vitro*. Our data on *Trem2* expression are consistent with a previous study reporting suppressed *Trem2* in response to intraperitoneal LPS injection (Zheng et al., 2016). These changes in expression of *Trems* occurred alongside marked induction of both *Nos2* and *Arg1* (Figure 7A). We also determined the effects of acute focal transient cerebral ischaemia induced by MCAO, a model causing a prominent inflammatory response and microglial reactivity. *Trem1* expression was markedly increased and *Trem2* expression significantly suppressed in ipsilateral brain homogenates 24 h after induction of MCAO (Figure 7B). In addition to microglial reactivity, bone marrow-derived myeloid cells, which can express *Trem1*, infiltrate the brain after acute cerebral ischaemia. Therefore, to assess induction specifically in microglia in this model, we used flow cytometry to selectively identify microglia using their characteristic CD11b^+^CD45^*lo*^Ly6C^−^ expression profile and measured TREM1 expression intensity on this population (Figure 7C). Expression of TREM1 was significantly increased on microglia in MCAO compared to sham-operated mice confirming induction of microglial TREM1 *in vivo*.

## Discussion

Our findings in the present study provide new insight to the regulation of *TREM* family expression in microglia. Key findings include (1) the divergent regulation of the major TREM family receptors, TREM1 and TREM2, under neuroinflammatory conditions *in vitro* and *in vivo*, (2) the common role for NF-κB in controlling the divergent responses, and (3) that in the context of TLR stimulation, the regulatory profile varies according to TLR isoform.

We found marked differences in microglial gene expression of the different *Trem* receptors in the steady-state mouse brain. Expression of *Trem2* was abundant (as indicated relative to established highly expressed microglial genes) in all forebrain regions examined in contrast to *Trem1* and *Trem3*. This pattern may reflect the involvement of TREM2 in homeostatic microglial function such that expression and signaling through the TREM2 pathway are important constitutively for normal microglial function. Indeed, the discovery of mutations in TREM2 that impair basic microglial physiological activities such as phagocytosis support the critical role for TREM2 for homeostatic function (Takahashi et al., 2005; Kleinberger et al., 2014). In contrast, TREM1 and TREM3 (mouse only), may play more prominent roles when induced under inflammatory conditions as amplifiers of cytokine responses (Sharif and Knapp, 2008) therefore negligible expression in steady-state microglia is consistent with their quiescent inflammatory phenotype. Our data showed that *Trem2* expression was highly enriched in purified microglia compared to brain homogenates which supports microglia as the predominant and possibly sole cellular source of *Trem2* in the CNS. Although previous studies have shown immunoreactivity for TREM2 protein on other cells (Giuseppina et al., 2004; Guerreiro et al., 2013b) it is possible this could reflect detection of the soluble ectodomain fragment bound to ligand.

The expression profiles of both *Trem1* and *Trem2* in response to stimulation were similar in all *in vitro* systems used and showed marked alterations to LPS but not IL-4 (with or without IL-13) stimulation. IL-4, a key mediator of alternative activation, caused marked induction of the archetypal marker *Arg1* indicating cells were generally responsive to IL-4. A previous study showed induction of TREM2 protein by IL-4 stimulation in peritoneal macrophages (Turnbull et al., 2006) suggesting that there could be differences in TREM2 regulation in microglia and macrophages. Nonetheless, we also do not exclude that IL-4 in combination with other ligands *in vitro* or *in vivo* might drive TREM2 expression. The contrasting induction of *Trem1* and suppression of *Trem2* in response to LPS challenge was striking and is comparable with the profiles described in myeloid cells outside the brain, including macrophages and dendritic cells (Bouchon et al., 2000; Turnbull et al., 2006; Ito and Hamerman, 2012). We also observed similar expression responses in two *in vivo* models of CNS inflammation, intracerebral LPS injection and focal cerebral ischaemia induced by MCAO. The comparable *in vivo* responses to LPS challenge and MCAO are interesting but not surprising given that sterile inflammation induced by ischaemic hypoxia and cell death trigger the release of damage-associated molecular patterns (DAMPs) or “alarmins” that activate similar receptors to microbial-derived ligands, including TLRs (Rock et al., 2010). TREM1 amplifies TLR-induced cytokine responses and its expression is induced in response to a number of infectious stimuli (Bouchon et al., 2001; Netea et al., 2006; Ornatowska et al., 2007; Hommes et al., 2014). In contrast, TREM2 deficiency in macrophages caused aggravated cytokine responses (Hamerman et al., 2006; Turnbull et al., 2006). Thus, the responses of *Trem1* and *Trem2* we show *in vitro* and *in vivo* highlight the possibility of co-ordinated but divergent regulation of their expression as a potent mechanism to tune the strength of microglial inflammatory responses. Moreover, the potent suppression of *Trem2* suggests certain acute inflammatory or injurious conditions in the CNS could impair TREM2-dependent microglial physiological functions. We restricted our analysis to <48 h after challenge, thus the extent to which changes we report are sustained or reversible are unknown (our unpublished data suggests normalization of expression does occur for both *Trem1* and *Trem2* several days later).

The signaling mechanisms underlying the regulation of microglial TREM expression and whether these mechansims share common elements is not well understood. Here we show that NF-κB is a common transcriptional regulator involved in LPS-induced microglial alterations to both *Trem1* and *Trem2* supporting the co-ordinated nature of their divergent responses. Consistent with this effect on *Trem1* expression, the *Trem1* promoter contains consensus binding sites for NF-κB among other inflammation-induced transcription factors and in macrophages NF-κB was shown to bind to the *Trem1* promoter (Zeng et al., 2007). The rapid and potent induction of *Trem1* we observed after LPS challenge or ischaemia is consistent with the well-established and rapid activation of NF-κB that can occur within 5 min of stimulation (Lei et al., 2014). In contrast, the *Trem2* promoter does not contain NF-κB consensus sites and given the normal role of NF-κB is to increase the rate of transcription it is unlikely a direct interaction between NF-κB and *Trem2* mediates the suppressive effects of LPS or cerebral ischaemia. A possible mechanism could be through NF-κB-induced micro RNAs (miRNAs) that can negatively regulate gene expression through binding the 3′ untranslated region (UTR) of target genes and inducing mRNA degradation or inhibiting translation (Valencia-Sanchez et al., 2006). Recent studies have identified miRNA-34a as an NF-κB-inducible miRNA upregulated in the diseased brain and this induction correlated with suppressed TREM2 expression (Alexandrov et al., 2013; Zhao et al., 2013). Further work showed that miRNA-34a could interact directly with the 3' UTR of the TREM2 mRNA (Bhattacharjee et al., 2016). We noted that the induction of *Trem1 in vitro* occurred as early as 2 h after LPS stimulation whereas the suppression of *Trem2* was not observed until 6 h. These kinetics are consistent with a rapid and direct transcriptional regulation of *Trem1* and a suppressive mechanism on *Trem2* involving an intermediate such as a miRNA and subsequent mRNA degradation. Our data demonstrating the non-uniform effects of stimulating different TLR subtypes on microglial *Trem1* and *Trem2* provides further insight to their regulation. Of particular note is that *Trem1* was induced only by ligands (LPS and poly I:C) that activate TLRs capable of signaling via the MYD88-independent TRIF module (i.e., TLR3 and TLR4) (O'Neill et al., 2013). In contrast, *Trem2* was suppressed by ligands activating all TLRs we tested including those that can signal via MYD88 only (TLR2/1, TLR9), MYD88 and TRIF (TLR4) or TRIF only (TLR3) although suppressive effects were weakest for TLR3. Both the MYD88 and TRIF pathways can activate NF-κB thus in agreement with the common effects of NF-κB pathway inhibition we found. However, the TLR sensitivity profiles suggest possible differences for *Trem1* and *Trem2* regulation in signaling upstream of NF-κB. Induction of *Trem1* appears to be dependent on the MYD88-independent TRIF pathway whereas suppression of *Trem2* could involve either MYD88 or TRIF modules, although further work targeting individual subcomponents of TLR signaling is needed to definitively assess this. Given the interest in immunomodulatory therapeutics targeting TLR signaling (Parker et al., 2005), it may be important to consider that effects on relative *Trem1* and *Trem2* expression could be variable depending on the specific elements of TLR signaling targeted.

TREM2 mutations have been associated with several neurodegenerative diseases (Colonna and Wang, 2016) and TREM1 variants with cognitive decline and Alzheimer-related amyloid pathology (Replogle et al., 2015). Most, although not all, TREM2 mutations appear to be loss-of-function raising the possibility of augmenting activity in the TREM2 pathway (e.g., via synthetic ligands or activating antibodies) as a potential therapeutic strategy in certain circumstances. Indeed, lentiviral overexpression of TREM2 reduced pathology and improved cognitive performance in a mouse model of AD-like amyloidosis (Jiang et al., 2014). However, the efficacy of pharmacological approaches may depend on the availability of the transmembrane receptor, which will be influenced by transcriptional regulation among other factors. Although our present study was limited to acute neuroinflammatory conditions, our data nonetheless highlight the importance of understanding how the more chronic neuroinflammatory environment in the aging and degenerating brain might influence TREM expression. It is pertinent to note that TREM2 expression was reduced in hippocampus from AD patients compared to controls (Zhao et al., 2013), although increased TREM2 expression has also been shown in AD-like mouse models and models of demyelinating disease (Piccio et al., 2007; Cantoni et al., 2015; Jay et al., 2015; Wang et al., 2015). Thus, a clearer understanding of how TREM expression is regulated in age-related neurological disease will be needed to guide how best to apply TREM-targeted interventions.

## Author contributions

BM conceived study; BM, RO, KG, CD, AA designed and performed mouse studies and analyzed data; JA, LH designed and performed human studies and analyzed data; BM, LH wrote manuscript.

## Funding

This work was supported by grants from MRC (MR/L003384/1) and BBSRC (BB/J004332/1) and PhD scholarships from the Darwin Trust of Edinburgh (KG) and BBSRC (CD). The Roslin Institute and Edinburgh Genomics are partly supported through core grants from NERC (R8/H10/56), MRC (MR/K001744/1) and BBSRC (BB/J004243/1, BB/J004332/1).

### Conflict of interest statement

The authors declare that the research was conducted in the absence of any commercial or financial relationships that could be construed as a potential conflict of interest.
