# Corrigendum: Divergent Neuroinflammatory Regulation of Microglial TREM Expression and Involvement of NF-κB

**DOI:** 10.3389/fncel.2017.00256

**Published:** 2017-08-24

**Authors:** Rosie Owens, Kathleen Grabert, Claire L. Davies, Alessio Alfieri, Jack P. Antel, Luke M. Healy, Barry W. McColl

**Affiliations:** ^1^The Royal (Dick) School of Veterinary Studies, University of Edinburgh Midlothian, UK; ^2^Neuroimmunology Unit, Montreal Neurological Institute, McGill University Montreal, QC, Canada; ^3^UK Dementia Research Institute, University of Edinburgh, Edinburgh Medical School Edinburgh, United Kingdom

**Keywords:** microglia, neuroinflammation, myeloid cells, NF-κB, TREM2, lipopolysaccharide (LPS), microglial activation

In the published article, there was an error regarding the affiliation[s] for Barry W. McColl. As well as having affiliation 1, they should also have the affiliation “UK Dementia Research Institute, University of Edinburgh, Edinburgh Medical School, Edinburgh, United Kingdom.” The authors apologize for this error and state that this does not change the scientific conclusions of the article in any way.

## Conflict of interest statement

The authors declare that the research was conducted in the absence of any commercial or financial relationships that could be construed as a potential conflict of interest.

